# Respiratory burst function of ovine neutrophils

**DOI:** 10.1186/1471-2172-10-25

**Published:** 2009-05-08

**Authors:** John-Paul Tung, John F Fraser, Peter Wood, Yoke Lin Fung

**Affiliations:** 1 Australian Red Cross Blood Service, Research and Development Laboratory, Kelvin Grove, Queensland, 4059, Australia; 2 The University of Queensland, School of Medicine (Central Clinical Division), Queensland, 4029, Australia; 3 Critical Care Research Group, The Prince Charles Hospital, Chermside, Queensland, 4032, Australia; 4 Princess Alexandra Hospital, Haematology Department, Woolloongabba, Queensland, 4102, Australia

## Abstract

**Background:**

Respiratory burst function resulting in the release of reactive oxygen species such as superoxide anion (O_2_^-^) from neutrophils is one of the key mechanisms of the innate immune system, and maladaptive control of this mechanism is thought to play a pivotal role in the development of pathologies such as acute lung injury and sepsis. Ovine models of these pathologies are limited by the poor understanding of ovine neutrophil respiratory burst function.

**Results:**

Aspects of ovine neutrophil respiratory burst function to be characterised were: i) the maximum rate of O_2_^- ^generated (*V*_*max*_); ii) the time taken to reach *V*_*max*_; iii) the total amount of O_2_^- ^generated during the reaction; and iv) the duration of the reaction. As well as for unstimulated neutrophils, these aspects were also characterised after incubation with a priming agonist (platelet activating factor [PAF], tumour necrosis factor alpha [TNF-α] and lipopolysaccharides [LPS]) activating agonists (N-formylmethionyl-leucyl-phenylalanine [fMLP] and phorbol 12-myristate 13-acetate [PMA]) or a combination of a priming and an activating agonist. In the absence of priming or activating agonists, ovine neutrophils displayed a low level of respiratory burst function which was not enhanced by either PAF, TNF-α, LPS or fMLP, but was significantly enhanced by PMA. The PMA-induced respiratory burst function was further enhanced by pre-incubation with PAF, but not with TNF-α or LPS. By varying the length of pre-incubation with PAF it was demonstrated that this effect decreased as the duration of pre-incubation with PAF increased, and that PAF was enhancing PMA's effects rather than PMA enhancing PAF's effects.

**Conclusion:**

This study successfully adapted a commonly used method of measuring human neutrophil respiratory burst function to characterise different aspects of ovine neutrophil respiratory burst function. This improved understanding of ovine neutrophils will facilitate the validitation of ovine biomedical models of human pathologies in which neutrophils have been implicated.

## Background

Neutrophils comprise one of the major cellular components of the innate immune system. They are rapidly recruited to sites of infection and their response includes phagocytosis of the injurious element, the release of pre-formed granular enzymes and proteins and the *de novo *production of a range of potentially damaging but ephemeral, reactive oxygen species (ROS) such as superoxide anion (O_2_^-^) [1]. ROS are generated by a variety of intracellular mechanisms, although the predominant mechanism, referred to as respiratory burst, is based upon the assembly and activation of Nicotinamide Adenine Dinucleotide Phosphate (NADPH) oxidase which then catalyses the univalent reduction of molecular oxygen (O_2_) to O_2_^- ^[1-4].

In healthy individuals this process is closely regulated as the inappropriate release of ROS by neutrophils can cause damage to the surrounding tissue and is thought to be a key factor in the development of pathologies such as acute lung injury (ALI) and its most severe form, acute respiratory distress syndrome (ARDS), as well as the multiple organ failure characteristic of sepsis. As the scope for investigating these pathologies in humans is limited, the development of relevant in-vivo animal models is crucial in attempting to elucidate the mechanisms by which they develop. Ovine models have been extensively used in the study of ALI/ARDS [5-13] and sepsis [14-22], however they are limited by a lack of understanding of the ovine neutrophil which has been less well studied than its human counterpart. Due to the neutrophil's central role in innate immunity and its postulated role in pathologies such as ALI/ARDS and sepsis, a better understanding of ovine neutrophils is crucial to improving the validity of these ovine biomedical models as well as improving the understanding of immunology and pathology in sheep.

Respiratory burst function provides a key measure of neutrophil function, and studies investigating this have used different techniques such as luminometry, fluorometry, precipitation reactions and photometry [23], some of which have been adapted to study ovine neutrophil respiratory burst function [23-38]. Photometric assays based upon the superoxide dismutase- (SOD) inhibitable reduction of cytochrome c have been well standardised for the measurement of human neutrophil respiratory burst function. Aspects such as the agonists used and their concentrations and incubation times, as well as which parts of the human neutrophil respiratory burst function that the assays measure are of importance, with the standard agonists being Platelet Activating Factor (PAF) and N-formylmethionyl-leucyl-phenylalanine (fMLP), and the maximum rate of O_2_^- ^release (*V*_*max*_) as the standard measure [1,39-42].

There have been several studies that have used photometric methods to investigate the respiratory burst function of neutrophils isolated from the blood of healthy sheep [24,26,28,29,34,37,43], however differences in assay methodology, agonists, and in the units measured have hindered comparisons between the studies. The relevance of results from these studies has also been hampered by the technical limitations of the assay protocols used. Stopping the cytochrome c reduction by cold-fixing the cells [24,37] or simply by not taking any further readings [28,29] means that these assays do not measure the reaction reaching its natural completion, and may bias the assay towards faster reacting agonists. The technique of measuring SOD-inhibitable reduction of cytochrome c has not been utilised to characterise the ovine neutrophil respiratory burst response to biologically relevant agonists such as lipopolysaccharides (LPS), the purified endotoxin from *Escherichia coli *which is used in sheep models of sepsis [14-16], and the inflammatory cytokine, Tumour Necrosis Factor-alpha (TNF-α), which is released from monocytes and tissue macrophages in response to LPS, PAF and other agonists [44,45]. Priming, a process in which the response of neutrophils to an activating agonist is enhanced by prior exposure to a priming agonist, has been well-characterised in human neutrophils [1,37,46-57], but with the exception of Buchta's paper [24], there has been little investigation into the effects that combinations of agonists have on ovine neutrophil respiratory burst function.

This paper aimed to take a standard human neutrophil respiratory burst assay [58,59] and adapt it for the measurement of ovine neutrophil respiratory burst function. This assay would also be adapted to measure not just the maximum rate of the reaction (*V*_*max*_), but also the total amount of O_2_^- ^generated during the reaction, the length of the reaction and the time taken to reach *V*_*max *_thus allowing us to better characterise neutrophil respiratory burst function. This modified assay could then be used to define changes in ovine neutrophil respiratory burst function in response to a range of agonists, and by using these agonists both individually and in combination, it was possible to determine if sheep neutrophils demonstrate a response analogous to the priming and activation demonstrated in human neutrophils.

## Results

### Neutrophil isolation

Whole blood collected from sheep had an average neutrophil count of 2.70 ± 3.50 × 10^9 ^cells/L (average ± standard deviation). The average neutrophil yield of the isolations was 35.21 ± 16.76% of starting numbers of neutrophils, with an average purity of 83.81 ± 8.70% neutrophils in the total leucocyte population, an average erythrocyte contamination of 0.004 ± 0.007 × 10^12 ^cells/L, and an average platelet contamination of 19.89 ± 22.59 × 10^9 ^cells/L.

### Assessment of respiratory burst stimulation by a single agonist

The respiratory burst function of isolated ovine neutrophils was assessed with either a priming or an activating agonist or with a control (nil) in which buffer only was used, and these results are shown in Table 1. Maximum rate (*V*_*max*_) results are reported as nmoles O_2_^- ^per 2 × 10^5 ^neutrophils per minute, and results for total O_2_^- ^are reported as nmoles O_2_^- ^per 2 × 10^5 ^neutrophils. Unstimulated neutrophils had a low level of respiratory burst function, with an average *V*_*max *_of 0.216 ± 0.113 nmol O_2_^-^/2 × 10^5 ^neutrophils/min (average ± SD) and an average total O_2_^- ^of 1.884 ± 1.527. This result indicates that the cells were not activated during isolation or testing, and that resting ovine neutrophils display low levels of respiratory burst function.

**Table 1:** Characteristics of ovine neutrophil respiratory burst function in response to a range of priming and activating agonists

	**n**		**V**_*max*_ (nmoles O_2_^-^/2 × 105 neutrophils/min)	**time to *V*_*max*_** (min)	**total amount of O_2_^- ^released** (nmoles O_2_^-^/2 × 10^5 ^neutrophils)	**duration of reaction** (min)
**Nil**	29	mean	0.216	16.0	1.885	65.5***
		std dev	0.113	6.9	1.527	33.8

**PAF** **(20 μM)**	28	mean	0.558	11.3**	2.657	27.5
		std dev	0.308	23.3	1.956	21.0

**TNF-α** **(10 ng/mL)**	6	mean	0.339	5.3	1.255	72.5
		std dev	0.212	7.2	0.484	38.2

**LPS** **(50 ng/mL)**	6	mean	0.650	8.7	1.106	44.2
		std dev	0.362	5.8	0.441	32.3

**PMA** **(20 ng/mL)**	26	mean	1.221***	14.4	24.340***	58.7
		std dev	0.532	7.3	4.264	20.5

**fMLP** **(20 μM)**	28	mean	0.567	5.2***	1.688	36.4**
		std dev	0.288	5.2	1.452	31.5

Ovine neutrophils (2 × 10^5^) were incubated either with buffer only (Nil) or one of a range of priming (PAF, TNF-α, LPS) and activating agents (PMA, fMLP). Total assay length was 120 minutes. Parameters were measured as described in Methods. Asterisks represent level of significance compared to unstimulated cells (Nil); * p < 0.05; ** p < 0.01; *** p < 0.001.

When incubated individually with ovine neutrophils each of the priming agonists: PAF, TNF-α, and LPS displayed low levels of respiratory burst function with *V*_*max *_values of 0.558 ± 0.307 nmol O_2_^-^/2 × 10^5 ^neutrophils/min, 0.340 ± 0.213 nmol O_2_^-^/2 × 10^5 ^neutrophils/min, and 0.652 ± 0.363 nmol O_2_^-^/2 × 10^5 ^neutrophils/min respectively; and with total O_2_^- ^values of 2.657 ± 1.955 nmol O_2_^-^/2 × 10^5 ^neutrophils, 1.257 ± 0.485 nmol O_2_^-^/2 × 10^5 ^neutrophils, and 1.105 ± 0.443 nmol O_2_^-^/2 × 10^5 ^neutrophils respectively. This did not represent a significant increase in respiratory burst function compared to unstimulated ovine neutrophils for any of the agonists (P > 0.05 for both *V*_*max *_and total O_2_^- ^for each of the agonists).

Of the activating agonists phorbol 12-myristate 13-acetate (PMA) produced the strongest response with an average *V*_*max *_of 1.222 ± 0.532 nmol O_2_^-^/2 × 10^5 ^neutrophils/min and an average total O_2_^- ^of 24.340 ± 4.264 nmol O_2_^-^/2 × 10^5 ^neutrophils. This represented a significant increase in respiratory burst function compared to unstimulated ovine neutrophils (P < 0.001 for both *V*_*max *_and total O_2_^-^). Analyses of the time taken to reach *V*_*max *_(14.38 ± 7.30 min) and the length of the reaction (58.66 ± 20.55 min) showed that the PMA-induced respiratory burst was a slow reaction with a long duration. The other activating agonist, fMLP, produced a weaker response with an average *V*_*max *_of 0.567 ± 0.288 nmol O_2_^-^/2 × 10^5 ^neutrophils/min and an average total O_2_^- ^generated of 1.688 ± 1.453 nmol O_2_^-^/2 × 10^5 ^neutrophils. This did not represent a significant increase in respiratory burst function compared to unstimulated ovine neutrophils (P > 0.05 for both *V*_*max *_and total O_2_^- ^generated).

### Combinations of agonists

**Figure 1:**
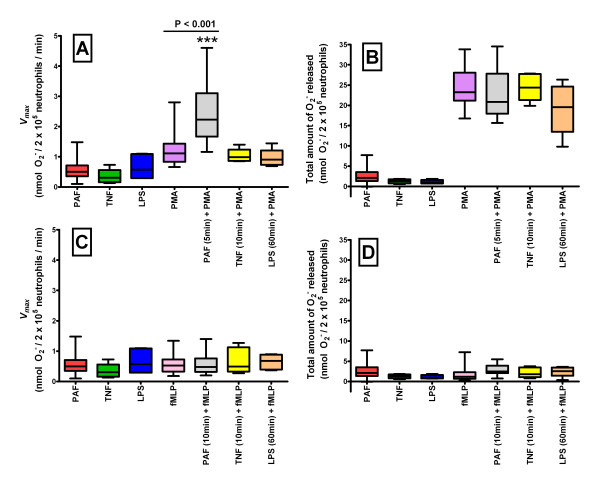
**A comparison of the effects on ovine neutrophil respiratory burst function when incubated with either a single agonist or with a combination of agonists**. Ovine neutrophils were incubated with either a single agonist or with a priming agonist (PAF, TNF-α, and LPS incubations were for 5, 10 and 60 minutes respectively) and an activating agonist (either PMA in A and B, or fMLP in C and D). *V*_*max *_(A and C) and the total amount of O_2_^- ^released (B and D) are shown. In A and B, n = 27, 6, 6, 26, 18, 6 and 5 respectively. In C and D, n = 27, 6, 6, 28, 20, 6 and 5 respectively. Results are shown in the form of a Box and Whiskers graph, and groups were compared using one way ANOVA followed by *post hoc *Bonferroni test for multiple comparisons. Significance was determined at P < 0.05.

In order to investigate the ability of combinations of agonists to enhance the respiratory burst function of ovine neutrophils, cells were incubated firstly with a priming agonist before the subsequent addition of an activating agonist. PMA was added following a 10 minute incubation with either PAF or TNF-α, or following a 60 minute incubation with LPS (Fig 1A and 1B), and fMLP was added after a 10 minute incubation with either PAF or TNF-α, or after a 60 minute incubation with LPS (Fig 1C and 1D).

Compared to ovine neutrophils that were incubated with PMA alone (1.222 ± 0.532 nmol O_2_^-^/2 × 10^5 ^neutrophils/min [Fig 1A]), there was a significant increase in *V*_*max *_(P < 0.001) when the cells were first incubated with PAF for 5 minutes prior to the subsequent addition of PMA (2.408 ± 0.888 nmol O_2_^-^/2 × 10^5 ^neutrophils/min [Fig 1A]). There was, however no significant change in total O_2_^- ^generated (from 24.340 ± 4.264 nmol O_2_^-^/2 × 10^5 ^neutrophils for PMA by itself to 23.000 ± 5.752 nmol O_2_^-^/2 × 10^5 ^neutrophils for PAF followed by PMA; P > 0.05; [Fig 1B]). Analyses of the time taken to reach *V*_*max *_(8.14 ± 6.20 min) and the duration of the reaction (41.58 ± 24.09 min) showed no significant change (P > 0.05 in both cases) when compared to PMA alone.

The PMA-induced respiratory burst response of ovine neutrophils showed no significant changes in either *V*_*max *_or total O_2_^- ^generated (P > 0.05 in both cases) when the cells were incubated with TNF-α for 10 minutes (*V*_*max*_: 1.028 ± 0.202 nmol O_2_^-^/2 × 10^5 ^neutrophils/min [Fig 1A]; total O_2_^- ^generated: 24.490 ± 3.161 nmol O_2_^-^/2 × 10^5 ^neutrophils [Fig 1B]) or with LPS for 60 minutes (*V*_*max*_: 0.956 ± 0.293 nmol O_2_^-^/2 × 10^5 ^neutrophils/min [Fig 1A]; total O_2_^- ^generated: 19.160 ± 6.276 nmol O_2_^-^/2 × 10^5 ^neutrophils [Fig 1B]) relative to cells that received PMA only. There were also no significant changes (P > 0.05 in both cases) in the time taken to reach *V*_*max *_or in the duration of the reaction relative to cells stimulated with PMA alone (data not shown).

The fMLP-induced respiratory burst response of ovine neutrophils showed no significant changes in either *V*_*max *_or total O_2_^- ^generated (P > 0.05 in all cases) when the cells were incubated with PAF for 10 minutes (*V*_*max*_: 0.765 ± 0.956 nmol O_2_^-^/2 × 10^5 ^neutrophils/min [Fig 1C]; total O_2_^- ^generated: 2.797 ± 1.404 nmol O_2_^-^/2 × 10^5 ^neutrophils [Fig 1D]), TNF-α for 10 minutes (*V*_*max*_: 0.650 ± 0.389 nmol O_2_^-^/2 × 10^5 ^neutrophils/min [Fig 1C]; total O_2_^- ^generated: 4.893 ± 6.751 nmol O_2_^-^/2 × 10^5 ^neutrophils [Fig 1D]) or with LPS for 60 minutes (*V*_*max*_: 0.648 ± 0.247 nmol O_2_^-^/2 × 10^5 ^neutrophils/min [Fig 1C]; total O_2_^- ^generated: 2.458 ± 1.281 nmol O_2_^-^/2 × 10^5 ^neutrophils [Fig 1D]) relative to cells that received fMLP only (*V*_*max*_: 0.567 ± 0.288 nmol O_2_^-^/2 × 10^5 ^neutrophils/min [Fig 1C]; total O_2_^- ^generated: 1.688 ± 1.453 nmol O_2_^-^/2 × 10^5 ^neutrophils [Fig 1D]). There were also no significant changes (P > 0.05 in all cases) in the time taken to reach *V*_*max *_or in the duration of the reaction relative to cells stimulated with fMLP alone (data not shown).

### Enhanced Respiratory Burst Function?

**Figure 2:**
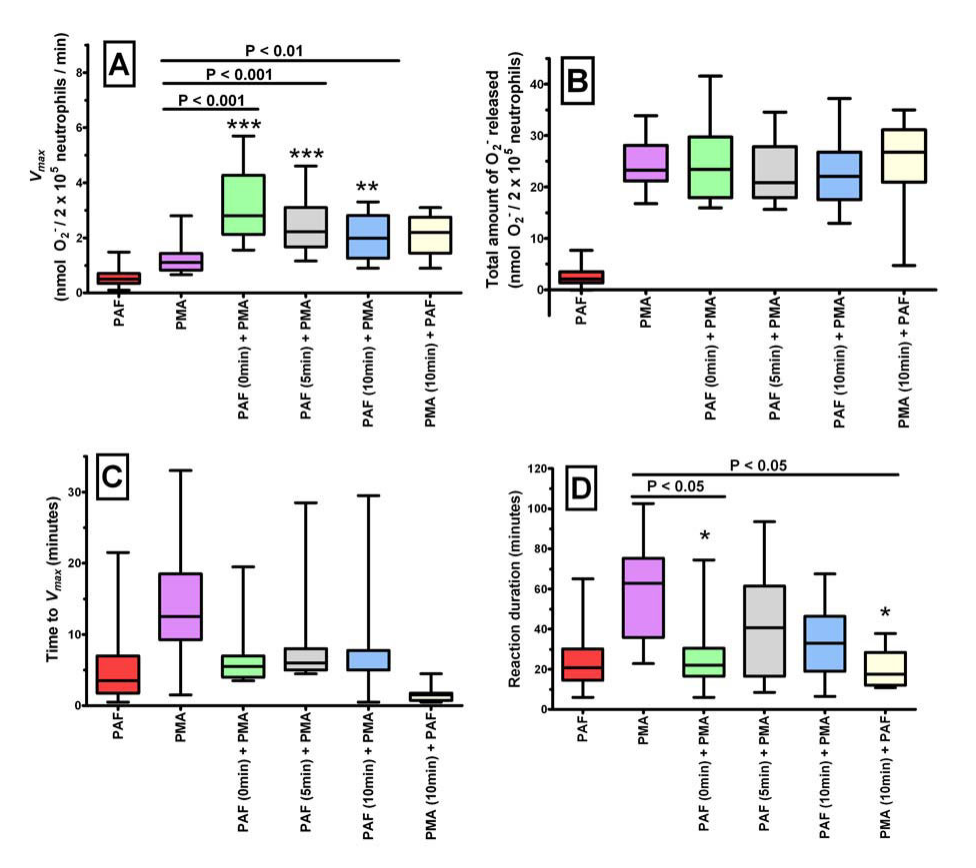
**A comparison of the effects on PMA-induced ovine neutrophil respiratory burst function when varying the length of PAF incubation, and the order of agonist addition**. Ovine neutrophils were incubated with either a single agonist (PAF or PMA; n = 27 and n = 26 respectively) or with a combination of PAF and PMA. Incubation times investigated were 0 minutes (simultaneous addition of PAF and PMA; n = 19), and both 5 and 10 minutes (PAF followed by PMA; n = 18 and n = 21 respectively). The order of addition was also varied, with PMA being added first, incubated for 10 minutes, and then PAF being added (n = 8). Results are shown for *V*_*max *_(A), time taken to reach *V*_*max *_(B), the total amount of O_2_^- ^released (C), and reaction duration (D). Results are shown in the form of a Box and Whiskers graph, and groups were compared using one way ANOVA followed by *post hoc *Bonferroni test for multiple comparisons. Significance was determined at P < 0.05.

In order to investigate whether incubation of ovine neutrophils with PAF causes an enhanced respiratory function following subsequent PMA stimulation, the duration of incubation was varied and the order of addition was reversed (i.e. PMA incubation and then addition of PAF) and results are shown in Figure 2.

When ovine neutrophils were incubated with PAF for 10 minutes prior to the addition of PMA there was an average *V*_*max *_of 2.010 ± 0.775 nmol O_2_^-^/2 × 10^5 ^neutrophils/min (Figure 2A) and an average total O_2_^- ^generated of 22.950 ± 6.619 nmol O_2_^-^/2 × 10^5 ^neutrophils (Figure 2B). When ovine neutrophils were incubated with both PAF and PMA simultaneously (i.e. 0 min incubation) there was an average *V*_*max *_of 3.202 ± 1.327 nmol O_2_^-^/2 × 10^5 ^neutrophils/min (Figure 2A) and an average total O_2_^- ^generated of 24.990 ± 7.658 nmol O_2_^-^/2 × 10^5 ^neutrophils (Figure 2B). When the order of addition was reversed, and ovine neutrophils were incubated with PMA for 10 minutes prior to the addition of PAF there was an average *V*_*max *_of 2.100 ± 0.812 nmol O_2_^-^/2 × 10^5 ^neutrophils/min (Figure 2A) and an average total O_2_^- ^generated of 24.660 ± 9.634 nmol O_2_^-^/2 × 10^5 ^neutrophils (Figure 2B).

Figure 2A shows that the strongest change in *V*_*max *_is observed when PAF and PMA are simultaneously added to ovine neutrophils, with the magnitude of the response decreasing as the time interval between the two agonists is increased. Compared to the response to PMA alone, there is a significantly enhanced response in *V*_*max *_for the simultaneous addition (P < 0.001) or after either 5 minutes (P < 0.001) or 10 minutes (P < 0.01), but not when the order was reversed (P > 0.05). Figure 2B shows that there were no significant changes in the total amount of O_2_^- ^generated for ovine neutrophils stimulated by PMA when comparing cells stimulated by PMA only with those that were also stimulated with PAF. Figure 2C shows that there was a decrease in the time taken to reach *V*_*max *_when ovine neutrophils were incubated with PAF and PMA (in any combination) as compared to PMA alone, however this was not significant (P > 0.05 in all cases). As shown in Figure 2D, a significant decrease in the duration of the reaction compared to PMA alone (58.66 ± 20.55 min) was observed when PAF and PMA were added simultaneously and when the order was reversed (28.13 ± 18.38 min and 20.61 ± 10.19 min respectively; P < 0.05 in both cases), but not when PAF was incubated for either 5 or 10 minutes before the addition of PMA (41.58 ± 24.09 min and 33.18 ± 16.52 min respectively; P > 0.05 in both cases).

## Discussion

The ability of neutrophils to destroy bacteria or other pathogens is dependant upon their ability to mount an effective respiratory burst response. The respiratory burst function is commonly investigated by assays based upon the SOD-inhibitable reduction of cytochrome c by O_2_^-^. Changes to the respiratory burst function of isolated human neutrophils induced by a range of priming and activating agonists has been well characterised, however the respiratory burst function of ovine neutrophils has been less well characterised. There have been fewer reports of ovine neutrophil respiratory function, and these studies are limited due to either: the methodology used, the small number of agonists investigated, or the lack of investigation into combinations of agonists. This study aimed to address these limitations by adapting a standardised method of investigating human neutrophil respiratory burst function to investigate the changes in ovine neutrophil respiratory burst function due to incubation with a range of agonists, both individually and in combination.

In previous studies of neutrophil respiratory burst function the maximum reaction rate or *V*_*max *_has been the principal measure [1,39,40,58,59]. The current study collected data on *V*_*max*_, but has additionally collected data on the time taken to reach *V*_*max*_, the total amount of O_2_^- ^released during the reaction and the duration of the reaction. This additional data allowed a more complete characterisation of the ovine neutrophil respiratory burst function. The assay used in this study was run for a two hour period as compared to the 10 – 60 minute time period that previous studies were run for [24,28,34,37]. This extra time proved to be important because it allowed the reactions to run to completion, and allowed sufficient time for the priming periods previously shown to be optimal for these agonists [1]. The reaction duration and time taken to reach *V*_*max *_data obtained in this study for PMA-induced ovine neutrophil respiratory burst function (Table 1) indicates that in Buchta's study [24] when these reactions had been stopped at 10 minutes, the reaction would not have been completed, and would not have reached *V*_*max*_. This would indicate that Buchta's study may have underestimated the PMA-induced ovine neutrophil respiratory burst function. Since the reactions in this study were allowed to run to completion, with regular readings taken throughout the assay, the data presented here is likely to be more accurate.

In this study the agonists considered to be priming agonists for human neutrophils (PAF, TNF-α, and LPS) did not induce a significant change in ovine neutrophil respiratory burst function when added individually. This result is consistent with those from studies of human neutrophil respiratory burst function showing that only some priming agonists are capable of inducing a significant response of their own, and even then this is only when they are added in high concentrations that may not be physiologically relevant [1,60]. Of the two agonists considered to be activating agonists for human neutrophils (PMA and fMLP), when added individually only PMA induced any significant change in ovine neutrophil respiratory burst function relative to unstimulated cells, with titrations showing that this response was dose-dependent (data not shown). The pathways by which PMA and fMLP activate neutrophil respiratory burst function are different. Studies of human neutrophils have shown that PMA stimulates the signalling element, protein kinase c (PKC) directly whereas fMLP does so indirectly by binding to G-protein linked receptors on the surface of the neutrophil that subsequently dissociate and activate multiple signalling pathways including mitogen -activated protein kinase (MAPK) and lipid kinases, as well as phospholipases which produce the second messengers responsible for activation of PKC [1,2,4].

In humans it has been shown that while some priming agonists cause an increase in fMLP receptor expression, the receptor-generated response is generally not the rate-limiting step in the priming and activation response and so this does not cause any increased response [3,4,47]. Previous reports have shown that fMLP has a negligible effect on ovine neutrophil respiratory burst function, and have hypothesised that this is due to either the low expression or the absence of receptors for this peptide on the surface of the cell [24,37], so it is possible that this may be a reaction-limiting step in ovine neutrophils. This study therefore investigated the possibility that incubation of ovine neutrophils with a priming agonist prior to the subsequent addition of fMLP would increase the expression of fMLP receptors to a level at which ovine neutrophil respiratory burst function to fMLP could be measured. The incubation periods and the concentrations used in this study demonstrated that neither PAF, TNF-α, or LPS was capable of enhancing ovine neutrophil respiratory burst function in response to fMLP. This provides evidence supporting the hypothesis that fMLP receptors are not expressed on the surface of ovine neutrophils, however to prove this hypothesis conclusively, ovine neutrophil surface expression studies using anti-fMLP receptor antibodies would be required.

Studies into human neutrophil respiratory burst function have demonstrated that priming agonists act to increase the response to PMA although the exact mechanisms by which they act remains uncertain and are thought to vary for different agonists. PAF is thought to act by enhancing PKC activity via a calcium-dependent mechanism [60] whereas TNF-α priming has been demonstrated to be independent of extracellular calcium [48] and priming of PMA-induced respiratory burst activity by LPS has not yet been demonstrated. Based upon these differences in priming mechanisms for the priming agonists used in this study, the finding that only PAF appeared to affect the PMA-induced respiratory burst function of ovine neutrophils was not unexpected nor was it novel.

This study demonstrated that there was a significant increase in *V*_*max *_for ovine neutrophils incubated with PAF followed by PMA or when they were added simultaneously but not when incubated with PMA followed by PAF, however this did not correlate with any significant change in the total amount of O_2_^- ^released during the reactions. In other words, the total amount of O_2_^- ^released in response to PMA stimulation remained constant regardless of PAF co-stimulation. It would make sense that if the total amount of O_2_^- ^released during the reaction remains constant while the *V*_*max *_increases, then the duration of the reaction should decrease, however this was found to be the case only when PAF and PMA were added simultaneously and not when there PMA was added after a 5 or 10 minute incubation with PAF. A possible explanation for this is that the magnitude of PAF-priming of the PMA-induced respiratory burst function of ovine neutrophils is at its maximum either immediately or shortly after PAF addition, and then decreases rapidly over the 10 minute time period tested here.

This study has provided evidence that PAF is capable of having an enhanced effect upon the subsequent PMA-induced respiratory burst function of ovine neutrophils. Firstly the *V*_*max *_values observed for ovine neutrophils stimulated by both PAF and PMA was not just greater than that of either agonist alone but also was greater than the combined *V*_*max *_values obtained by simply adding together the effects of each agonist alone. Additionally, the *V*_*max *_data presented here indicates that PAF is enhancing PMA-induced respiratory burst function rather than PMA enhancing PAF-induced respiratory burst function. Further evidence, such as that from blocking studies using PKC inhibitors or PAF antagonists, would be required to better elucidate the relationship between PAF and PMA and to ascertain whether this is analogous to the priming and activation described for human neutrophils.

These experiments have demonstrated that LPS and TNF-α did not enhance either fMLP- or PMA-induced ovine neutrophil respiratory burst function. This is not unexpected because even for human neutrophils the PMA-induced respiratory burst function has not been demonstrated to be primed by LPS, and the evidence from this and other studies [24,37] indicates that there is no significant fMLP-induced ovine neutrophil respiratory burst function. So while this study has not been able to demonstrate priming by either LPS or TNF-α, further studies using alternative activating agonists may demonstrate whether LPS and TNF-α are capable of priming ovine neutrophil respiratory burst function. Alternatively the investigation of specific surface molecule markers of neutrophil priming and activation would provide a measure of ovine neutrophil priming and activation independent of respiratory burst function, and this different technique may also demonstrate whether LPS and TNF-α are capable of priming ovine neutrophils.

Studies to define the role of endogenous PAF in ovine biomedical models have trialled PAF-receptor antagonists with mixed results [61,62], so an alternative method of assessing this would be beneficial. While PMA is not a biologically relevant agonist, its characterisation here and in other studies [24,34] has demonstrated that it is useful as an in-vitro activating agonist for the ovine neutrophil respiratory burst function. The possibility that PAF may be capable of enhancing or priming the ovine neutrophil respiratory burst function for subsequent activation by PMA is an important finding because it means that it may be possible to investigate endogenous PAF priming of ovine neutrophils by isolating them and stimulating them with PMA. It has been demonstrated in humans that neutrophils isolated from injured or sick patients display a reduced respiratory burst function in response to priming and activating agonists as compared to healthy controls [58,59], so by using the assay described in this study with the agonists PAF and PMA, it may be possible to investigate whether neutrophils isolated from sick or injured sheep display a similar reduced respiratory burst function. This would greatly enhance the relevance of ovine biomedical models of human pathologies such as ALI/ARDS and sepsis. Another way to enhance these models would be to directly compare the expression of key markers linked to neutrophil priming and activation on the surfaces of both human and ovine neutrophils.

## Conclusion

This study has described an assay based upon the SOD-inhibitable reduction of cytochrome c to measure several aspects of ovine neutrophil respiratory burst function. The actions of several well-characterised human neutrophil priming and activating agonists were investigated both individually and in combination. It was shown that the priming agonists PAF, TNF-α and LPS did not on their own induce the ovine neutrophil respiratory burst. It was demonstrated that fMLP, both on its own, and after priming by either PAF, TNF-α and LPS, did not induce the ovine neutrophil respiratory burst. PMA, however was capable of inducing the ovine neutrophil respiratory burst by itself, and this response was significantly enhanced by pre-incubation with PAF, with an increase in *V*_*max *_and a decrease in the duration of the reaction. These changes indicate that PAF in some way, possibly analogous to the priming described for human neutrophils, enhances the PMA-induced ovine neutrophil respiratory burst. The findings of this study advance our knowledge and understanding of ovine neutrophils, and improve the validity of ovine biomedical models of human pathologies such as ALI/ARDS and sepsis in which neutrophils have been implicated.

## Methods

### Preparation of reagents

To minimise the likelihood of isolated neutrophils becoming inadvertently primed or activated during isolation or testing all the solutions and materials used in this study were sterile or endotoxin-free. Cytochrome C, SOD, PAF, LPS, TNF-α, Phorbol 12-Myristate 13-Acetate (PMA) and fMLP were obtained from Sigma (Castle Hill, Australia) and were diluted into stock solutions that were stored at -80°C. Working solutions were obtained on the day of assay by dilution of stock solutions in Hanks' buffered salt solution containing calcium, magnesium and glucose (HCMG; Gibco, Invitrogen, Auckland, New Zealand).

### Animals and sample collection

This study has been approved by the University Animal Ethics Committee (The University of Queensland, Brisbane, Australia). The sheep used in this study were Merino ewes, housed in outdoor pens. Fifty-six blood samples (25 – 100 mL) from 38 different sheep were collected from either the right or left jugular vein. Blood samples were collected into syringes and immediately transferred into sodium citrate tubes (Becton Dickinson, Macquarie, Australia).

### Isolation of ovine neutrophils

Neutrophils were isolated from fresh ovine whole blood in a method adapted from previously published methods of ovine and human neutrophil isolation [1,24,34,37]. Whole blood was centrifuged at 400 g for 30 min. Platelet rich plasma was discarded, and the buffy coat and packed cell volume was pooled. Erythrocytes were lysed by hypotonic lysis with 10 mL packed cells gently mixed with 36 mL water for 20 seconds before isotonicity was restored with the addition of 4 mL of 9% sodium chloride (Ajax Fine Chemicals, Taren Point, Australia). Leucocytes were recovered following centrifugation at 600 g for 5 min, washed three times in Hanks' buffered salt solution (HBSS; Invitrogen, Auckland, New Zealand), and then recovered in HBSS. This leucocyte preparation was then underlaid with equal volumes of Ficoll Paque™ Plus (GE Healthcare, Uppsala, Sweden) and then Mono-Poly Ficoll-Hypaque Resolving Medium (MP Biomedicals, LLC, Ohio, USA), and centrifuged at 600 g for 20 minutes. After centrifugation two leucocyte layers were visible at the interfaces between the buffer, the Ficoll Paque™ Plus, and the Mono-Poly Ficoll-Hypaque Resolving Medium, with the upper layer containing mononuclear leucocytes and any remaining platelets, and the lower layer containing granulocytes, of which the majority will be neutrophils. Remaining erythrocytes form a pellet at the bottom of the tube. The lower layer of neutrophils was collected and then washed three times in HBSS with centrifugation at 600 g for 5 minutes, and then was finally re-suspended in HBSS.

### Assessments of purity and yield

Aliquots of ovine whole blood and isolated neutrophils were taken and an automated full blood count and white blood cell differential was performed using the Abbott Cell-Dyn 3200 Multiparameter Haematology Analyser (Abbott Diagnostics Division, North Ryde, Australia). Purity was assessed based upon the percentage of neutrophils in the total leucocyte population of the isolation, as well as the levels of erythrocyte and platelet contamination. Yields were calculated by dividing the number of neutrophils in the final isolation by the number of neutrophils in the volume of whole blood collected, and were expressed as a percentage of the starting numbers. This method aimed for and achieved leucocyte purity of greater than 80% neutrophils, with erythrocyte and platelet contamination below 0.05 × 10^12^/L and 0.5 × 10^9^/L respectively, and with a yield of greater than 30%.

### Assessment of respiratory burst function

The assay used to assess respiratory burst function was modified from that described by Fung et al. and was based upon the SOD-inhibitable reduction of cytochrome c at 550 nm in microtitre plates [2,4,54]. For each agonist or combination of agonists, duplicates were tested with a single well containing SOD as a control. Neutrophils (2 × 10^5^) were added to a reaction mixture containing HCMG, and cytochrome c (50 uM) and then incubated at 37°C with shaking for 2 hours on the Bio-Rad 680 plate reader (Bio-Rad Laboratories, Hercules, California, USA). Measurements of optical density (OD) at 550 nm were taken at 30 second intervals by the plate reader. After an initial 10 minute incubation period agonists (PAF, TNF-α, LPS, fMLP, and PMA) were added to the appropriate wells, and the assay continued for a total of 2 hours. In wells where combinations of agonists were investigated, the priming agonist was added first and then after a pre-determined incubation period the activating agonist was added, and the assay continued until a total time of 2 hours had been reached. The incubation times used were those suggested in the literature, except in the experiments to investigate the PMA-induced response to PAF incubation [1,58,59]. The average optical density from the duplicate wells less that of the control (SOD) well was then plotted against time.

**Figure 3:**
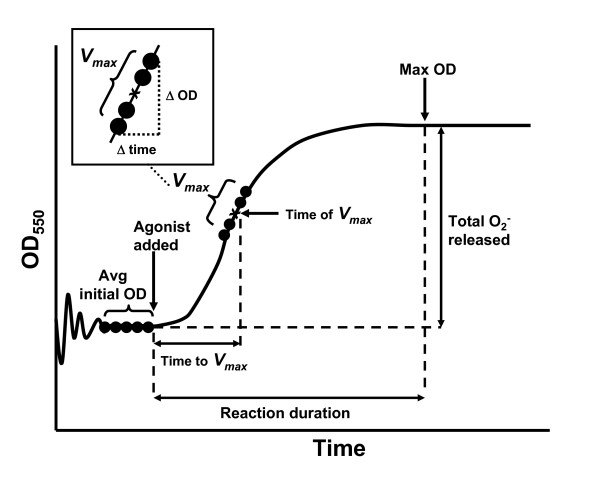
**An overview of methods of measuring ovine neutrophil respiratory burst function**. Ovine neutrophil respiratory burst function was characterised by *V*_*max*_, time to *V*_*max*_, the total amount of O_2_^- ^released and the reaction duration. *V*_*max *_was calculated from the 5 data-points corresponding to the steepest gradient of the graph as described in Materials and Methods. The time of *V*_*max *_was defined as the middle of these 5 data points, and the time taken to reach *V*_*max *_was calculated as the time interval between agonist addition and the time of *V*_*max*_. The average OD of the 5 data-points immediately preceding agonist addition was calculated, and the difference between this initial OD and the maximum OD reached in the reaction was used to calculate the total amount of O_2_^- ^generated in the reaction as described in Methods. The reaction duration was calculated as the time interval between agonist addition and the time when the maximum OD was reached.

Respiratory burst function was characterised as the maximal rate of O_2_^- ^generation (*V*_*max*_) and the total amount of O_2_^- ^generated. *V*_*max *_was calculated from the steepest slope with at least 5 data points using the molar extinction coefficient of 21.1 × 10^3 ^mol/L for ferricytochrome c, and was reported as nmoles O_2_^- ^per 2 × 10^5 ^neutrophils per minute (Figure 1). The total amount of O_2_^- ^generated was calculated from the difference between the average OD from the 5 data points preceding agonist addition and the maximum OD for that agonist, again using the molar extinction coefficient of 21.1 × 10^3 ^mol/L for ferricytochrome c, and was reported as nmoles O_2_^- ^per 2 × 10^5 ^neutrophils (Figure 3). Data was also collected for the duration of reaction, calculated as the time interval between the initial addition of an agonist and when the maximum OD of the reaction was reached, and the time to *V*_*max*_, calculated as the time interval between the addition of an agonist and when the reaction rate reached *V*_*max*_, with both of these values reported in minutes (Figure 3).

To decide upon concentrations of each agonist to use, different concentrations of the priming agonists (PAF, TNF-α, and LPS) were tested individually and in combination with a fixed concentration of each of the activating agonists (PMA at 20 ng/mL or fMLP at 20 μM). A concentration for each priming agonist was then chosen by taking into account the responses measured for both *V*_*max *_and the total amount of O_2_^- ^generated. The activating agonists were then tested at different concentrations both by themselves and with a fixed concentration of each of the priming agonists (20 μM of PAF, 10 ng/mL of TNF-α, and 50 ng/mL of LPS). In the same manner as for the priming agonists, a concentration for each activating agonist was then chosen by taking into account the responses measured for both *V*_*max *_and the total amount of O_2_^- ^generated. In these experiments there were cases where no significant response was evident regardless of the concentration of agonist used. When this occurred a concentration was chosen based upon the results of that agonist with PMA. In the case of fMLP, a small response was measured, and a dose was chosen based upon this small response. Subsequent statistical analysis revealed this response to not be significant.

### Statistical analyses

Where sufficient numbers of neutrophils were available assays were run with replicates (2–4 replicates), and an average value for each of the parameters measured was then calculated, otherwise assays were run in singlicate. All values were then imported in GraphPad Prism 4.03 for Windows (GraphPad Software, San Diego California, USA) and then the average values for each parameter as well as standard deviations and 95% confidence intervals were calculated. Results were then compared using one way ANOVA followed by *post hoc *Bonferroni test for multiple comparisons. Significance was determined at P < 0.05.

## Abbreviations

ALI: acute lung injury; ANOVA: analysis of variance; ARDS: acute respiratory distress syndrome; fMLP: N-formylmethionyl-leucyl-phenylalanine; HBSS: Hanks' buffered saline solution; HCMG: HBSS with calcium, magnesium and glucose; LPS: lipopolysaccharides; MAPK: mitogen-activated protein kinase; NADPH: nicotinamide adenine dinucleotide phosphate; O_2_: molecular oxygen; O_2_^-^: superoxide anion; OD: optical density; PAF: platelet activating factor; *Phox*: phagocyte oxidase; PI-3K: phosphoinositide-3 kinase; PKC: protein kinase C; PMA: phorbol 12-myristate 13-acetate; Rac2: ras-related C3 botulinum toxin substrate 2; ROS: reactive oxygen species; SD: standard deviation; SOD: superoxide dismutase; TNF-α: tumour necrosis factor alpha; *V*_*max*_: maximum rate of reaction.

## Authors' contributions

JPT was responsible for the conception, planning and performance of experiments, the interpretation of results, and for drafting this manuscript; YLF contributed to the conception, planning of experiments, interpretation of results and review of the manuscript. Both JFF and PW contributed to the conception of experiments, interpretation of results and the review of the manuscript.
